# Shedding light on sunscreen biosynthesis in zebrafish

**DOI:** 10.7554/eLife.07961

**Published:** 2015-05-12

**Authors:** Carolyn A Brotherton, Emily P Balskus

**Affiliations:** Department of Chemistry and Chemical Biology, Harvard University, Cambridge, United States; Department of Chemistry and Chemical Biology, Harvard University, Cambridge, United Statesbalskus@chemistry.harvard.edu

**Keywords:** vertebrates, gadusol, sunscreen, mycosporine, 2-epi-5-epi-valiolone synthase, biosynthesis, *E. coli*, *S. cerevisiae*, zebrafish

## Abstract

Zebrafish can synthesize a sunscreen compound called gadusol, which was previously thought to be acquired only through the diet.

**Related research article** Osborn A, Almabruk K, Holzwarth G, Asamizu S, LaDu J, Kean K, Karplus PA, Tanguay R, Bakalinsky A, Mahmud T. 2015. De novo synthesis of a sunscreen compound in vertebrates. *eLife* **4**:e05919. doi: 10.7554/eLife.05919

**Image** Species including fish, birds and reptiles have genes that may enable them to produce sunscreen molecules

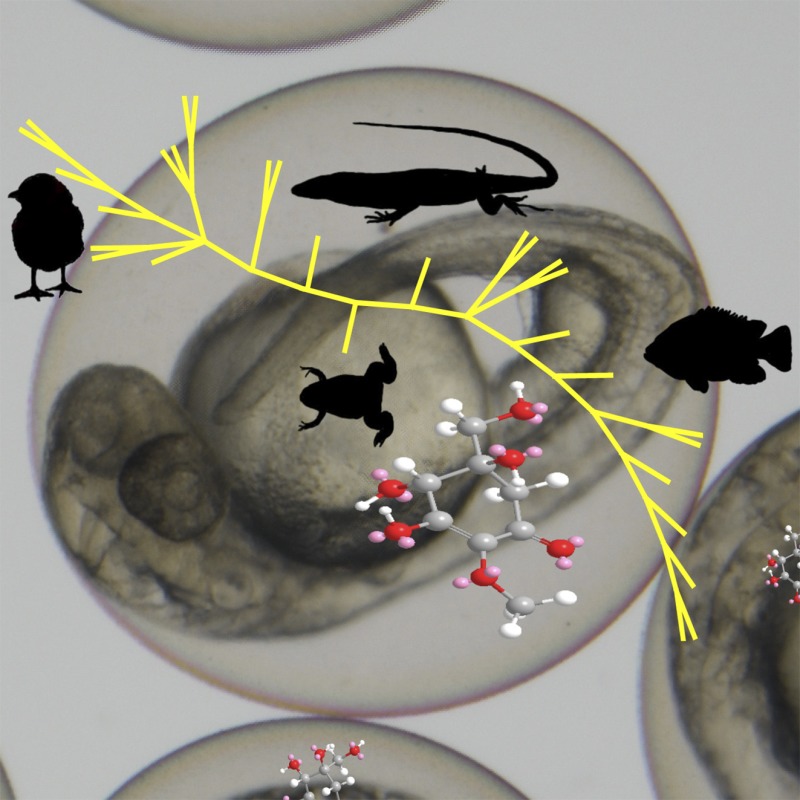

Just as many people apply sunscreen before they go to the beach, bacteria, fungi and algae that spend much of their time in the sun also need to protect themselves from solar radiation. These organisms make small molecules that act as sunscreens by absorbing harmful ultraviolet (UV) radiation. Small-molecule sunscreens—which include compounds called the mycosporine-like amino acids (MAAs) and a related compound called gadusol—are also found in corals, marine invertebrates and fish.

Sunscreen compounds like gadusol and the MAAs are important for the development, lifestyle and health of marine-dwelling organisms. For example, gadusol is found in high concentrations in the roes of fish, such as cod (Plack et al., 1981), as well as in sea urchin eggs (Chioccara et al., 1986), and it has been suggested that gadusol helps with embryonic development in these organisms. Sunscreen compounds also perform an array of roles in other organisms, including enhancing the UV-light vision of mantis shrimp (Bok et al., 2014).

It was thought for many years that the ability to synthesize small-molecule sunscreens was limited to microbes, and that higher marine organisms obtained these compounds exclusively from their diet. Now, in *eLife*, Taifo Mahmud and co-workers at Oregon State University—including Andrew Osborn, Khaled Almabruk and Garrett Holzwarth as joint first authors—show that zebrafish can synthesize gadusol (Osborn et al., 2015)*.* They also present evidence that the pathway used by zebrafish to make gadusol is distinct from the pathway used by microorganisms to synthesize MAAs. Moreover, they show that the gadusol biosynthetic genes are also found in the genomes of birds, reptiles and other organisms.

In microorganisms that biosynthesize MAAs, the biosynthetic pathway involves three core enzymes and uses a compound called sedoheptulose-7-phosphate as the starting material (Balskus and Walsh, 2010). The first step is performed by a dehydroquinate synthase-like enzyme. This enzyme is a member of a family of enzymes called the sugar phosphate cyclases, which catalyze the conversion of sugar molecules to products that contain a structure called a cyclohexane ring (Asamizu et al., 2012). Dehydroquinate synthase has a central role in the biosynthesis of aromatic amino acids, and is found in many branches of the tree of life—such as bacteria, archaea, fungi, plants and algae—but not in vertebrates.

Given the lack of sugar phosphate cyclases in vertebrates, the Oregon State team was surprised to find a gene that encoded a related enzyme in the genomes of fish, including zebrafish (*Danio rerio*), a well-known model organism. This gene was similar to the EEVS (short for 2-epi-5-epi-valiolone synthase) genes involved in the biosynthesis of bacterial natural products such as antibiotics and fungicides (Yu et al., 2005). The EEVS-like gene was clustered in the zebrafish genome with a gene of unknown function named MT-Ox (for methylation and oxidation). Surrounding these two genes were genes encoding several transcription factors that regulate essential cellular processes. The similarity of the EEVS-like gene to the genes that encode sugar phosphate cyclases in microbes raised the possibility that these zebrafish genes might be involved in the synthesis of related small molecules.

To explore this further, the team first expressed the zebrafish EEVS-like protein in *Escherichia coli* and incubated it alongside sedoheptulose-7-phosphate (the starting material used by microorganisms to make MAAs). The product of this reaction was 2-epi-5-epi-valiolone, which contains a cyclohexane ring. This confirms that the EEVS-like protein is a functional sugar phosphate cyclase.

The MT-Ox protein was then expressed in *E. coli* and incubated with its predicted cofactors and the 2-epi-5-epi-valiolone produced by the EEVS-like protein. This reaction produced a molecule that strongly absorbed UV light; structural studies revealed this was the small-molecule sunscreen gadusol. This work represents the first discovery of a gadusol biosynthetic pathway. Unexpectedly, the enzymes involved are distinct from those used by microbes to produce the closely related MAAs.

Having demonstrated that these two zebrafish enzymes could synthesize a small-molecule sunscreen, the Oregon State team next examined whether gadusol is actually produced in zebrafish*.* Quantitative reverse transcription-PCR analysis and organic extraction of zebrafish embryos showed that the gadusol biosynthetic genes are expressed in the developing embryo and that gadusol is indeed produced. These results clearly indicate that the gadusol found in zebrafish does not come from the dietary intake of this molecule. Instead, this sunscreen is biosynthesized from the primary metabolite sedoheptulose-7-phosphate by the activities of the EEVS-like and MT-Ox enzymes.

The team then used bioinformatics to examine whether the gadusol biosynthetic gene cluster occurs in other organisms, including algae, invertebrates and chordates. Unexpectedly, the gene cluster is found in many organisms that are not known to produce gadusol, including other fish, birds, reptiles and amphibians. However, it is not present in mammals or in marine animals called tunicates and lancelets. Furthermore, close relatives of these genes are not found in the genomes of non-vertebrate organisms, with two exceptions: a stramenopile and a microalgae. The EEVS-like genes from these two algal species are more similar to those found in vertebrates than those found in bacterial genomes. With these data in hand, the Oregon State team propose that the gadusol pathway identified in higher organisms could have come from a horizontal gene transfer event from an algal species to an ancestor of animals.

Overall, this work illuminates a novel pathway that constructs an important biological sunscreen, but it also raises a number of questions. How did this biosynthetic gene cluster evolve in animals, and why was it lost in some branches of the phylogenetic tree? Do the other animals that harbor the gene cluster actually produce gadusol, and what role does the biological sunscreen play in those organisms? Finally, why do marine animals that obtain MAAs from their diet also produce gadusol *de novo*? These lines of inquiry will likely reveal new roles for these intriguing small-molecule sunscreens.

**Figure 1.**
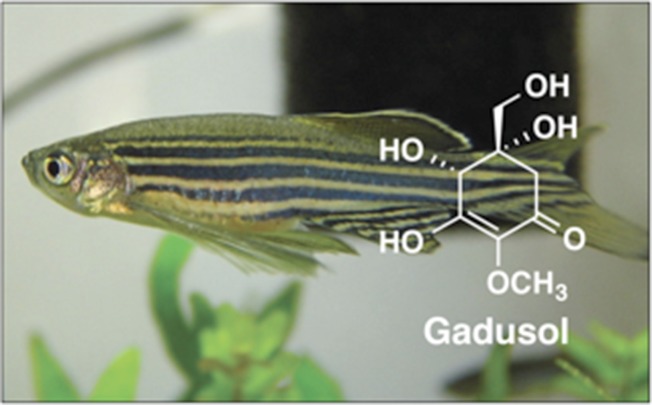
Many species produce small molecules that protect them from the sun by absorbing harmful ultraviolet radiation. The genome of the zebrafish (*Danio rerio*) contains a cluster of genes for a set of proteins that can make the sunscreen compound gadusol from sedoheptulose-7-phosphate. Microbes also use this molecule to make sunscreen compounds, but do so via a different pathway. The cluster of genes found in zebrafish is also present in several other vertebrates, including amphibians, reptiles and birds.

FIGURE CREDIT: Photograph taken by kamujp (CC BY-SA 2.0)

## References

[bib1] Asamizu S, Xie P, Brumsted CJ, Flatt PM, Mahmud T (2012). Evolutionary divergence of sedoheptulose 7-phosphate cyclases leads to several distinct cyclic products. Journal of the American Chemical Society.

[bib2] Balskus EP, Walsh CT (2010). The genetic and molecular basis for sunscreen biosynthesis in cyanobacteria. Science.

[bib3] Bok MJ, Porter ML, Place AR, Cronin TW (2014). Biological sunscreens tune polychromatic ultraviolet vision in mantis shrimp. Current Biology.

[bib4] Chioccara F, Zeuli L, Novellino E (1986). Occurrence of mycosporine related compounds in sea urchin eggs. Comparative Biochemistry and Physiology Part B: Comparative Biochemistry.

[bib5] Plack PA, Fraser NW, Grant PT, Middleton C, Mitchell AI, Thomson RH (1981). Gadusol, an enolic derivative of cyclohexane-1,3-dione present in the roes of cod and other marine fish. Biochemical Journal.

[bib6] Osborn A, Almabruk K, Holzwarth G, Asamizu S, LaDu J, Kean K, Karplus PA, Tanguay R, Bakalinsky A, Mahmud T (2015). De novo synthesis of a sunscreen compound in vertebrates. eLife.

[bib7] Yu Y, Bai L, Minagawa K, Jian X, Li L, Li J, Chen S, Cao E, Mahmud T, Floss HG (2005). Gene cluster responsible for validamycin biosynthesis in *Streptomyces hygroscopicus* subsp. *jinggangensis* 5008. Applied and Environmental Microbiology.

